# Active head-impulse tests may help detect acute unilateral vestibulopathy: a preliminary study

**DOI:** 10.3389/fneur.2026.1870852

**Published:** 2026-08-19

**Authors:** Sung-Hwan Kim, Euyhyun Park, Sun-Uk Lee, Jeong-Yoon Choi, Byung-Jo Kim, Ji-Soo Kim

**Affiliations:** 1 Department of Neurology, Korea University Medical Center, Seoul, Republic of Korea; 2 Department of Otorhinolaryngology-Head and Neck Surgery, Korea University College of Medicine, Seoul, Republic of Korea; 3 Neurotology and Neuro-ophthalmology Laboratory, Korea University Medical Center, Seoul, Republic of Korea; 4 Department of Neurology, Seoul National University College of Medicine, Seoul, Republic of Korea; 5 Dizziness Center, Clinical Neuroscience Center, Seoul National University Bundang Hospital, Seongnam, Republic of Korea; 6 BK21 FOUR Program in Learning Health Systems, Korea University, Seoul, Republic of Korea

**Keywords:** acute unilateral vestibulopathy, head-impulse test, nystagmus, vertigo, vestibular neuritis

## Abstract

**Introduction:**

Apart from conventional head-impulse tests (i.e., passive HITs), patency of the vestibulo-ocular reflex (VOR) can also be evaluated through self-generated HITs (i.e., active HITs). We aimed to evaluate the usefulness of active (self-generated) HITs in patients with acute unilateral vestibulopathy/vestibular neuritis (AUVP/VN) during the acute stages.

**Methods:**

We prospectively recruited 24 patients with AUVP/VN and 30 healthy participants from January 2019 to June 2026. All subjects underwent passive as well as active HITs. We compared the latency and amplitude of corrective saccades during active and passive HITs.

**Results:**

During active HITs, patients with AUVP/VN exhibited shorter latency (*p* < 0.001) and larger amplitude (*p* < 0.001) of their 1st corrective saccade compared with healthy participants. The latency of 1st corrective saccades during active HITs showed a positive correlation (*p* < 0.001), whereas the amplitude showed a negative correlation (*p* < 0.001) with the VOR gain during passive HITs. To differentiate patients with AUVP/VN from healthy participants, the sensitivity and specificity were 83.3 and 86.4%, respectively, at a cut-off latency of 242 ms for the 1st corrective saccade during active HITs with an area-under-the-curve (AUC) of 0.907. The sensitivity was 91.7% and the specificity was 93.3% at a cut-off value of 8.15° for amplitude of 1st corrective saccades, with an AUC of 0.971.

**Discussion:**

Detection of short-latency (≤ 242 ms) and large (≥ 8.15°) corrective saccades may help identify AUVP/VN during active HITs. Active HITs may provide complementary information for detecting acute unilateral vestibular hypofunction, although further validation is warranted.

## Introduction

Video head-impulse tests (vHITs) allow assessment of the vestibulo-ocular reflex (VOR) during rapid head rotations (1, 2). A deficient VOR is inferred from a decreased VOR response followed by a corrective catch-up saccades immediately after the head rotation (‘refixation saccade’). Unlike traditional caloric and rotatory tests, which can only evaluate the function of horizontal canals (HCs), vHITs enable assessment of each semicircular canal separately using physiologically relevant stimuli, with acceptable cost-effectiveness, and minimal inconvenience for the patient (3). Accordingly, video-HITs have become the forefront of vestibular evaluations.

Apart from conventional vHIT measures, earlier reports indicate that active HITs can also be used to assess VOR integrity: The VOR can be measured while the patient voluntarily generates head rotations in either direction while fixating on a distant, earth-fixed target. Indeed, patients with unilateral vestibular loss can occasionally present deficient VOR during these self-generated head movements (4–6). However, clinical application of active HITs has usually been limited since they often have normal VOR gain indistinguishable from healthy controls (4). Unlike passive HITs, the compensatory saccades occur mostly during head rotation (i.e., covert) during active HITs, and give a false calculation to show normal VOR gain (4, 7). Thus, analyses of VOR gain in active HITs are largely inaccurate in estimating vestibular function.

Notably, recent studies have demonstrated that active HITs can be used to differentiate peripheral vestibulopathy by analyzing the presence of corrective saccades (8, 9). Corrective saccades are observed in patients with chronic, profound vestibular deafferentation following translabyrinthine schwannoma surgery or congenital vestibular loss (8). The clinical utility of active HITs has not been adequately assessed in an acute clinical setting. In this study, we aimed to evaluate the usefulness of active (self-generated) HITs in patients with acute unilateral vestibulopathy/vestibular neuritis (AUVP/VN) during the acute stages, building upon recent findings in chronic vestibulopathy.

## Methods

### Patients and healthy participants

We prospectively recruited 50 consecutive patients with AUVP/VN from January 2019 to June 2026 at Korea University Medical Center. Among them, we excluded those in which the HITs were conducted more than 7 days after symptom onset (*n* = 15), poor cooperation or excessive eye blinks (*n* = 10), prior history of vestibular disorders (*n* = 4), and inferior vestibular neuritis (*n* = 1). Finally, 24 patients with AUVP/VN were included in the analyses (15 male; mean age ± standard deviation [SD] = 57 ± 17; range = 23–83 years; 10 with right-sided AUVP/VN). Healthy participants were recruited during the same period from those with no history of auditory or vestibular disorders (19 male; 52 ± 18 years; range = 24–80). To match age, healthy participants within an age range of ±5 years were selected for each patient. The diagnosis of AUVP/VN was made according to the diagnostic criteria proposed by the Barany society (10).

### Passive and active HITs

Passive HITs were performed for the HCs as described previously (11). Head and eye movements were recorded using video-based equipment (SLVNG, SLMED, Seoul, Republic of Korea) for quantitative measurement. The right eye position was recorded with a small, lightweight, high-speed digital video camera (Firefly MV, Point Grey Research Inc., Wilsonville, OR). The camera was mounted on a light plastic glasses frame with an elastic strap that locked the instrument tightly to the subject’s head. The image of the eye was reflected from an infrared mirror to the camera. The eye was illuminated by two infrared light-emitting diodes. Head velocity was measured using a three-axis gyroscope (L3G4200D, STMicroelectronics, Schiphol, The Netherlands). Peak acceleration and velocity were measured during passive and active head rotations. Video images were analyzed online to calculate eye position using a pupil-detection method based on a center-of-gravity algorithm written in C language. Eye velocity was obtained from data processed with a two-point differentiator and a low-pass filter (0–30 Hz bandwidth). The patient was seated 1.2 m in front of a target. Calibration of the eye position was carried out with a red dot sequentially presented from the center by 10 degrees in vertical and horizontal directions. Passive, unpredictable, high-acceleration, small-amplitude head rotations were delivered in the plane of the HCs. The VOR gains were measured for individual trials as the ratio of the mean velocity of the eye divided by the mean velocity of the head during a 40-ms window centered at the time of peak head acceleration. For passive HITs, abnormal VOR gain was defined as values outside the mean ± 2SD obtained from healthy participants (normal gains = 0.88–1.27) (12).

Then, the patient is instructed to make self-generated, ‘active’ horizontal head impulses on command while fixating on the same target. At least 10 consecutive head impulse data were selected, free of artifacts. Saccades were analyzed by two neurophysiologists blinded to clinical information (E. P. and J. Y. C.). The averaged values were calculated and used as statistical analysis.

**Figure 1:**
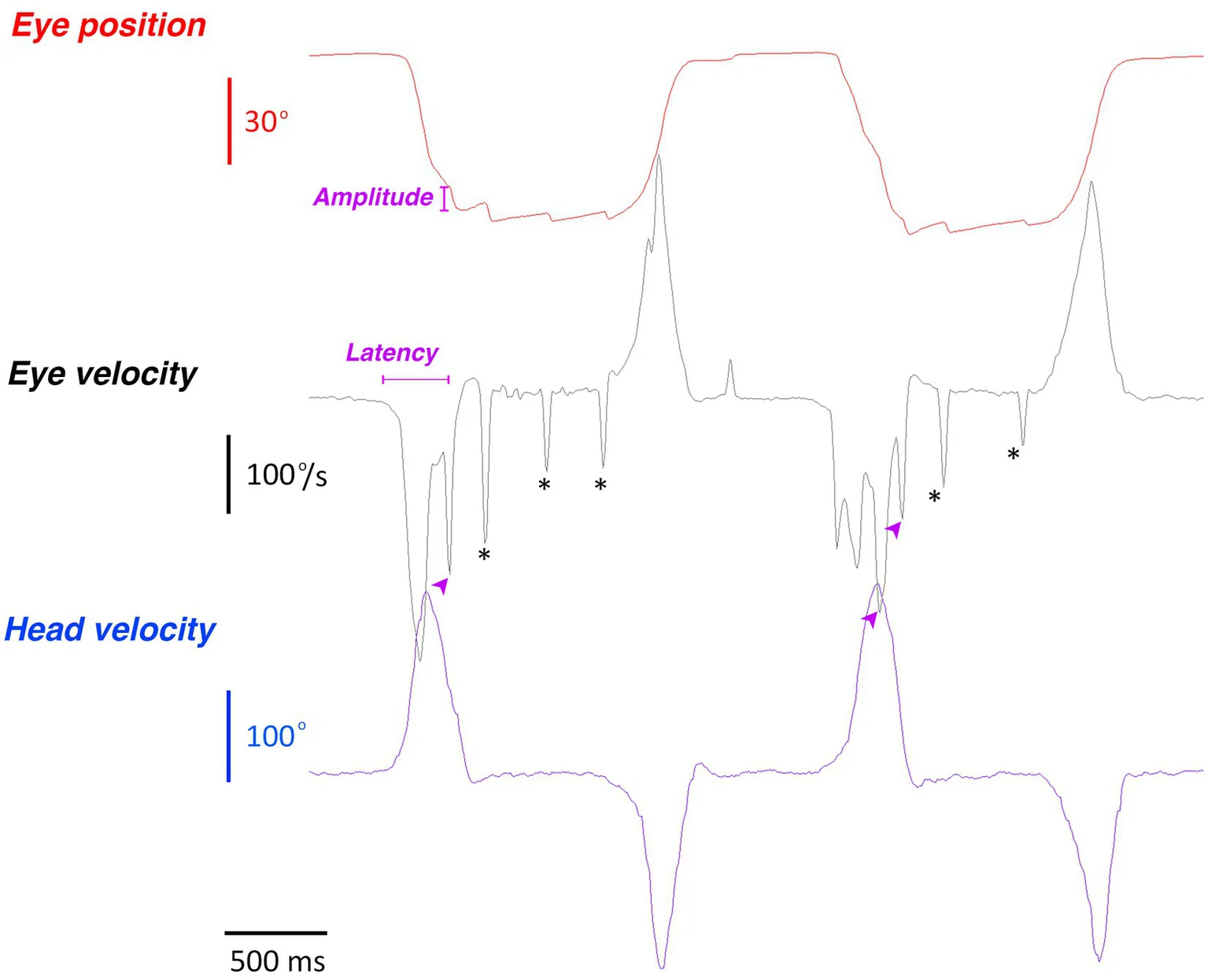
Analyses of corrective saccades in a patient with right vestibular neuritis as an example. Time series of eye position of the right eye (marked in red), the velocity of the right eye (marked in black), and the velocity of the head (marked in blue) in response to head impulses. The corrective saccades are collected only among fast eye movements that are out of phase of the main sequence of spontaneous nystagmus (asterisks). They are acknowledged only when the peak velocity exceeds 80°/s (2) the acceleration and deceleration flanks exceed 3,000°/s^2^ and (3) if the saccade duration lies within 10–80 ms. Please note the short-latency multiple corrective saccades (marked in purple arrowheads) that occur during the movement of the head.

The start of the head movement was defined as the point at which the head movement velocity exceeded 30°/s. Eye movements were defined as corrective saccades (1) if the eye peak velocity exceeded 80°/s, (2) if the acceleration and deceleration flanks exceeded 3,000 °/s^2^, and (3) if the saccadic duration was within 10–80 ms (8). The corrective saccades were determined only among those that were demarcated from the main sequence of spontaneous nystagmus (Figure 1). We measured the latency and amplitude of corrective saccades. Latency was defined as the interval from head movement to the peak velocity of corrective saccades. Saccadic amplitude was defined as the changes in eye position during corrective saccades. If there were multiple corrective saccades present during single impulses, each latency and amplitude were measured for the first, second, and third … corrective saccades, and those for the 1st corrective saccades were adopted for statistical analysis.

### Statistical analysis

Nominal variables were compared using χ ^2^ or Fisher’s exact test, and continuous variables using the Student t-test, paired t-test, and the Wilcoxon signed-rank test. A receiver operating characteristic (ROC) analysis was also conducted to decide the sensitivity and specificity of the continuous variables. Sensitivity and specificity were presented for cut-off values that maximize the Youden-Index [Sensitivity + (Specificity – 1)]. A bootstrap method was used to compare the area under the curve (AUC) for each variable, and DeLong’s test with Benjamini–Hochberg false discovery rate procedure was conducted for multiple pairwise comparisons. The 95% confidence intervals (CI) for sensitivity and specificity were estimated using the Wilson score method for binomial proportions.

To reduce the risk of optimism bias from deriving and evaluating ROC performance in the same dataset, we also performed stratified 5-fold cross-validation as an internal validation procedure. In each iteration, four folds were used as the training set to derive the optimal cut-off value using the Youden index, and the remaining fold was used as the test set. This process was repeated until each fold had served once as the test set. Cross-validated AUCs were calculated from the pooled out-of-fold predictions. Sensitivity and specificity were calculated using the training-derived cut-off values on the corresponding held-out test folds.

Statistical analyses were performed using the R software package (version 4.1.0; http://www.r-project.org), and the significance level was set at a two-tailed *p* < 0.05.

## Results

Cohen’s kappa values were 0.619 for active 1st saccadic latency, 0.740 for active 1st saccadic amplitude, 0.705 for passive 1st saccadic latency, and 0.882 for passive 1st saccadic amplitude for two independent neurophysiologists.

### Passive HITs

The VOR gains were decreased on the lesion side in 20 patients with AUVP/VN (20/24, 84%), ranging from 0.15 to 1.37 on the lesion side (mean ± SD = 0.61 ± 0.30). Four patients showed abnormal caloric tests despite normal VOR gains on HITs (4/24, 17%). Three patients (3/24, 13%) also showed a slight decrease in VOR gain on the healthy side (VOR gain range = 0.67–1.23, mean ± SD = 0.99 ± 0.17).

**Figure 2:**
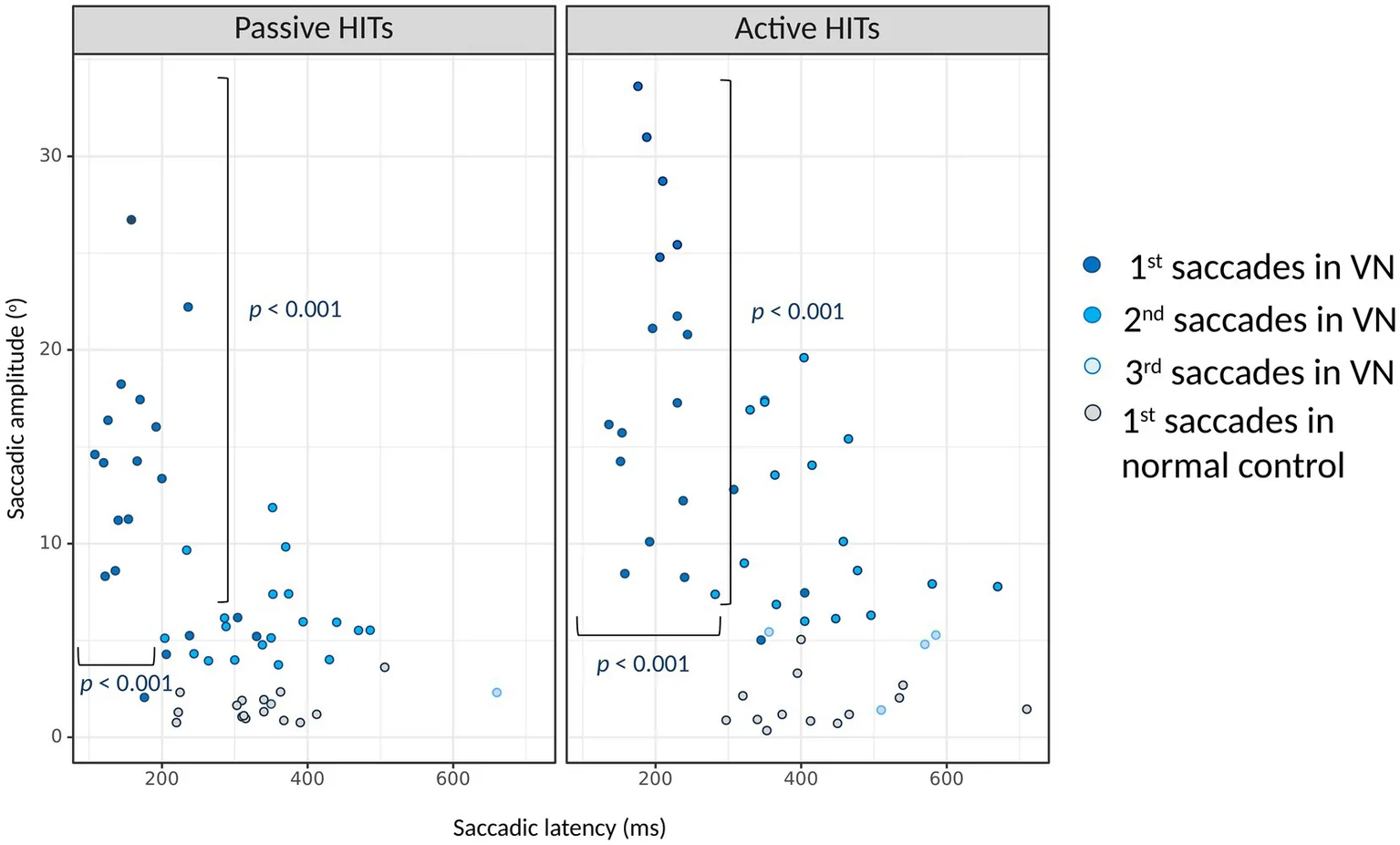
The latency of 1st corrective saccades is shorter in patients with vestibular neuritis (AUVP/VN) than in healthy participants during passive head-impulse tests (HITs, median [IQR] = 167 ms [144–192] vs. 341 ms [284–386], *p* < 0.001). The amplitude of 1st corrective saccades is larger in patients with AUVP/VN than in healthy participants (14.2° [10.8–16.8] vs. 0.9° [0.4–2.0], *p* < 0.001). During active HITs, the latency of 1st corrective saccades is shorter in patients with AUVP/VN than in healthy participants (187 ms [160–224] vs. 408 ms [324–548], *p* < 0.001). The amplitudes are larger in patients with AUVP/VN than in healthy participants (16.8° [12.3–24.1] vs. 1.8° [0.7–4.6], *p* < 0.001).

Corrective saccades were observed in all patients with AUVP/VN on the lesion side and in 27 healthy subjects on both sides (27/30, 90%). The latency of 1st corrective saccades was shorter in patients with AUVP/VN than in healthy participants (167 [144–192] vs. 332 ms [308–397], *p* < 0.001; Figure 2). Meanwhile, the amplitude of 1st corrective saccade was larger in patients with AUVP/VN than in healthy participants (14.2 [10.8–16.8] vs. 1.2° [0.5–1.8], *p* < 0.001, Figure 2). Twenty-two patients with AUVP/VN (22/24, 92%) showed multiple corrective saccades for each impulse on the lesion side (14 patients more than 3 times and 8 patients twice).

### Active HITs

Corrective saccades were observed in both directions in 14 patients with AUVP/VN (14/24, 58%). Corrective saccades were also found in 21 healthy participants on both sides (21/30, 70%). The latency of 1st corrective saccades was shorter in patients with AUVP/VN than in healthy participants (186.8 [159.5–224.4] vs. 407.9 [323.5–548.4] ms, *p* < 0.001, Figure 2). The amplitude of 1st corrective saccades was larger in patients with AUVP/VN than in healthy participants (16.8 [12.3–24.1] vs. 1.8° [0.7–4.57], *p* < 0.001, Figure 2). Seventeen patients with AUVP/VN (20/24, 83%) showed multiple corrective saccades for each impulse on the lesion side (12 for more than 3 times, and eight for twice).

The mean peak head acceleration ranged from 891 to 5,904°/s^2^ in patients with AUVP/VN and healthy participants. Low head acceleration (1,500°/s^2^) was observed in eight healthy participants (8/30, 27%) and three patients with AUVP/VN (3/24, 12.5%). Peak head acceleration (2,720.2 [2,062.2–3,057.7] vs. 2,581.3°/s^2^ [1,939.9–3,330.7], *p* = 0.900) did not differ between the lesion side and the healthy side in patients with AUVP/VN during active HITs. No differences were found in peak head acceleration (2,724.5 [1,493.2–3,674.0] vs. 2,586.9°/s^2^ [1418.3–3250.2], *p* = 0.141) between the right and left sides in healthy participants, either. The peak head acceleration did not differ between patients and healthy participants (2,720.2 [2,062.2–3,057.7] vs. 2,724.5°/s^2^ [1,493.2–3,674.0], *p* = 0.994 for lesion side; 2,581.3 [1,939.9–3,330.7] vs. 2,586.9°/s^2^ [1,418.3–3,250.2] for the healthy side). Head velocity also did not differ between the two groups (242.0 ± 72.0 vs. 230.1 ± 63.5°/s, *p* = 0.449 for lesion side; 239.6 ± 78.6 vs. 229.3 ± 65.8°/s, *p* > 0.999 for healthy side).

### Comparison between passive and active HITs

In patients with AUVP/VN, 1st saccadic amplitude was increased during active HITs than during passive HITs on the lesion side (*p* = 0.006), but not on the healthy side (*p* = 0.487; please refer to Table 1 for statistics). 1st saccadic latency was increased during active HITs than during passive HITs, both on the lesion side (*p* = 0.042) and healthy side (*p* = 0.006). The VOR gain did not differ between passive and active HITs on either the lesion side (*p* = 0.838) or the healthy side (*p* = 0.119). Peak head acceleration or velocity did not differ between passive and active HITs, either on the lesion side (*p* = 0.07 for peak head acceleration; *p* = 0.293 for peak head velocity) or healthy side (*p* = 0.064 for peak head acceleration; *p* = 0.276 for peak head velocity).

**Table 1:** Results of HITs in patients with AUVP/VN and healthy participants.

HIT parameters	AUVP/VN (*n* = 24)		*p* value for lesion vs. healthy	Healthy participants (*n* = 30)		*p* value for right vs. left
	Lesion side	Healthy side		Right	Left	
Passive HITs						
HC VOR gain	0.61 ± 0.30	0.98 ± 0.17	*< 0.001*	1.01 ± 0.12	0.98 ± 0.13	0.234
Peak head acceleration, °/s^2^	3,424.9 ± 967.8	3,438.6 ± 830.9	0.924	3,737.5 ± 642.3	3,869.4 ± 760.6	0.258
Peak head velocity, °/s	226 ± 37	222 ± 34	0.472	238 ± 26	241 ± 25	0.372
1st saccadic latency, s	0.17 [0.14–0.19]	0.26 [0.19–0.36]	*<0.001*	0.34 [0.28–0.39]	0.37 [0.32–0.44]	0.014
1st saccadic amplitude, °	14.2 [10.8–16.8]	1.5 [0.7–2.9]	*< 0.001*	0.93 [0.40–2.02]	1.4 [0.4–1.9]	0.974
Active HITs						
HC VOR gain	0.60 [0.45–0.76]	0.90 [0.74–1.07]	*0.002*	1.02 [0.95–1.05]	0.97 [0.90–1.06]	0.128
Peak head acceleration, °/s^2^	2,720.2 [2,062.2–3,057.7]	2,581.3 [1939.9–3330.7]	0.900	2,724.5 [1493.2–3674.0]	2586.9 [1418.3–3250.2]	0.141
Peak head velocity, °/s	246 [178–297]	222 [187–282]	0.686	243 [219–259]	240 [210–266]	0.802
1st saccadic latency, s	0.19 [0.16–0.22]	0.39 [0.33–0.46]	*<0.001*	0.41 [0.32–0.55]	0.45 [0.33–0.66]	0.562
1st saccadic amplitude, °	16.8 [12.3–24.1]	0.5 [0–2.5]	*<0.001*	0.7 [0.04–2.5]	0.5 [0.02–2.4]	0.670

AUVP/VN = acute unilateral vestibulopathy/vestibular neuritis, HC VOR = vestibulo-ocular reflex gain for horizontal canal, HITs = head-impulse tests, VOR = vestibulo-ocular reflex, °- degree.

In healthy participants, 1st saccadic latency was increased during active HITs compared to passive HITs on the right side (*p* = 0.039, but not on the left side *p* = 0.057; please refer to Table 1 for statistics). 1st saccadic amplitude did not differ during passive and active HITs, both for the right (*p* = 0.785) and left side (*p* = 0.721). The VOR gain did not differ between passive and active HITs on either the right (*p* = 0.957) or the healthy side (*p* = 0.897). Peak head acceleration was decreased during active HITs compared to passive HITs, either on the right (*p* = 0.012) and left side (*p* < 0.001). Peak head velocity did not differ between active and passive HITs either for the right (*p* = 0.765) or left side (*p* = 0.417).

### Correlation of active HITs with other vHIT parameters

**Figure 3:**
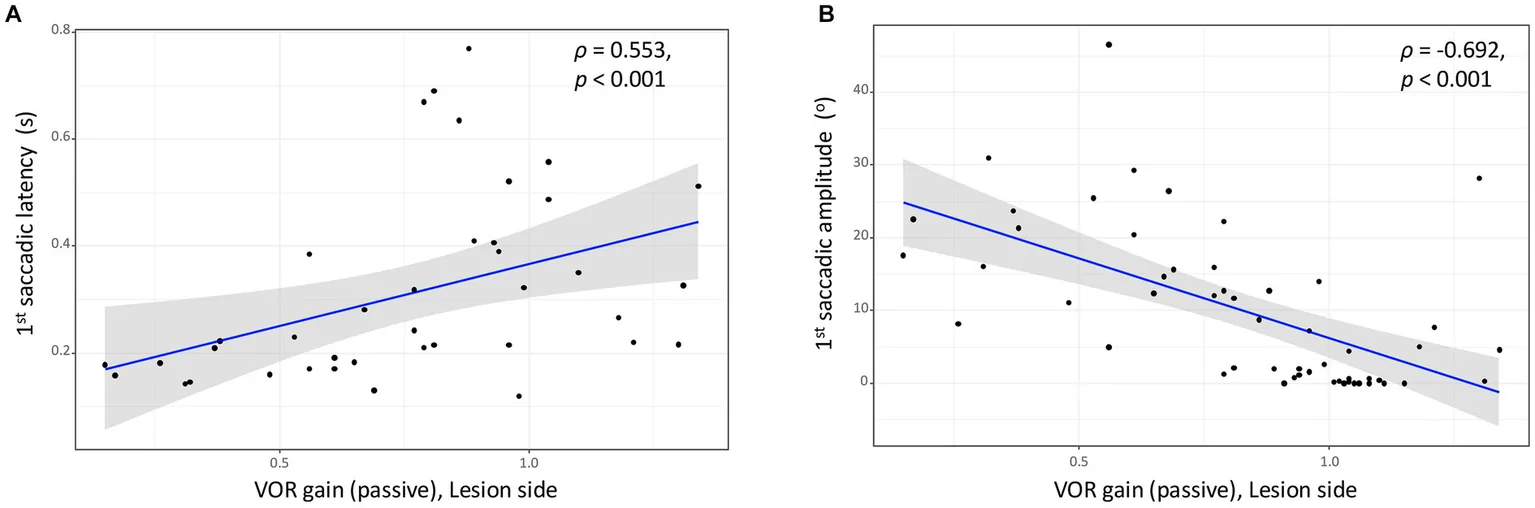
**(A)** The latency of 1st saccades during active head-impulse tests (HITs) shows a positive correlation with the vestibulo-ocular reflex (VOR) gain during passive HITs (*r* = 0.553, *p* < 0.001) on the lesion side. **(B)** The 1st saccadic amplitude shows a negative correlation with the VOR gain during passive HITs on the lesion side (*r* = −0.692, *p* < 0.001).

Latency of 1st corrective saccades during active HITs showed a positive correlation with the VOR gain during passive HITs (*ρ* = 0.553, *p* < 0.001, Figure 3A). In addition, the amplitude of 1st corrective saccades showed a negative correlation with the VOR gain during passive HITs (*ρ* = −0.692, *p* < 0.001, Figure 3B). The latency (*ρ* = 0.087, *p* = 0.565) and amplitude (*ρ* = 0.064, *p* = 0.644) of 1st corrective saccades did not show any correlation with the peak head accelerations during active HITs.

### ROC analysis

To differentiate patients with AUVP/VN from healthy participants, all variables showed at least good discriminatory power, with an AUC > 0.80 (Table 1). In particular, the sensitivity was 83.3% (95% confidence interval [CI] = 64.2–93.3), and the specificity was 86.4% (66.7–95.3) at a cut-off value of 242 ms for latency of 1st saccades during active HITs with an AUC of 0.907 (95% CI = 0.811–0.979). The sensitivity was 91.7% (74.2–97.7), and the specificity was 93.3% (78.7–98.2) at a cut-off value of 8.15° for the amplitude of 1st saccades during active HITs with an AUC of 0.989 (95% CI = 0.963–1.000). Pairwise comparisons of ROC curves using DeLong tests with Benjamini–Hochberg correction showed no significant differences among the AUCs of the tested parameters, including between the latency of the first saccades during active HITs and VOR gain during passive HITs (*q* = 0.780; Table 2).

**Table 2:** The discriminatory power of each parameter for vestibular neuritis (AUVP/VN) compared to healthy participants*.

Parameters	AUC (95% CI)	Cut-off value	Sensitivity (95% CI, %)	Specificity (95% CI, %)	vs. 1st saccadic latency (active)†	vs. 1st saccadic amplitude (active)†	vs. 1st saccadic latency (passive)†	vs. 1st saccadic amplitude (passive)†	vs. VOR gain (passive)†
1st saccadic latency (active), ms	0.907 (0.811–0.979)	≤242	83.3 (64.2–93.3)	86.4 (66.7–95.3)	—	0.350	0.463	0.333	0.780
1st saccadic amplitude (active), °	0.971 (0.926–0.997)	≥8.15	91.7 (74.2–97.7)	93.3 (78.7–98.2)	0.350	—	0.618	0.409	0.370
1st saccadic latency (passive), ms	0.949 (0.88–0.997)	≤254	91.7 (74.2–97.7)	89.3 (72.8–96.3)	0.463	0.618	—	0.350	0.409
1st saccadic amplitude (passive), °	0.989 (0.963–1.000)	≥7.63	91.7 (74.2–97.7)	100 (88.7–100)	0.333	0.409	0.350	—	0.350
VOR gain (passive)	0.897 (0.774–0.993)	≤0.77	79.2 (59.5–90.8)	100 (88.7–100)	0.780	0.370	0.409	0.350	—

^*^Values in the pairwise comparison columns are FDR-adjusted q-values from DeLong tests comparing AUCs.

^†^Values for the HIT parameters were calculated using the averaged measurements from the two independent evaluators.

°- degree.

AUC = area under the curve, CI = confidence interval, HIT = head-impulse test, VOR = vestibulo-ocular reflex.

For the internal validation analysis using stratified 5-fold cross-validation, the discriminatory performance of the HIT parameters remained robust. The cross-validated AUCs were 0.889 for active 1st saccadic latency and 0.965 for active 1st saccadic amplitude (Supplementary Table 1).

## Discussion

The findings of our study can be summarized as follows: First, patients with AUVP/VN exhibited shorter latency (≤ 242 ms) and larger amplitude (≥ 8.15°) of their 1st corrective saccade during active HITs compared to healthy participants. Second, the 1st saccadic amplitude was increased during active HITs compared with passive HITs in patients with AUVP/VN, a trend not replicated in healthy participants. Third, the VOR gain during passive HITs (which is conventionally used as a measure of VOR integrity) positively correlated with the latency, and negatively with the amplitude of 1st corrective saccades during active HITs. Collectively, the identification of short-latency, large-amplitude corrective saccades during active HITs can help differentiate AUVP/VN from healthy participants.

### Prior research on active HITs

Active HITs have been previously introduced, but their clinical significance has been questioned due to their low sensitivity (4, 9, 13): Corrective saccades mostly appeared smoothly during active head impulses, thereby making them indistinguishable from the VOR graph (4). Accordingly, unlike passive HITs, VOR gains were frequently normal during active HITs (4, 13). This false-negative result can be attributed to the low head acceleration during active HITs. Slow head rotation (< 100°/s) allows vestibular inputs from the contralateral canal or other ocular motor mechanisms to compensate for the deficient VOR, if any (14). Preprogramming and prediction may also enhance the VOR during voluntary head movements, masking the impairment (13, 15). In this context, the diagnostic yield of active HITs has reportedly been limited in clinical practice.

### Analysis of corrective saccades: an alternative for assessment of VOR patency

Measuring VOR gain is an intuitive method for quantifying VOR impairment in various vestibular disorders (3). Alternatively, detecting corrective saccades can provide an efficient estimate of VOR integrity (16). The 1st corrective saccade occurred mostly centered on 200 ms after active impulses, and corrective saccades are embedded during the head rotation (covert saccades). As our study replicated, measurements of VOR gain often fail to detect AUVP/VN. A time-series analysis of the individual impulse can identify these corrective saccades during active HITs (4). Our findings suggest that discriminating the 1st corrective saccade is important since only those with AUVP/VN give rise to short-latency (≤ 242 ms) and large (≥ 8.15°) corrective saccades during active HITs.

The mechanism of this corrective saccade is explained by the remaining vestibular function, somatosensory cues from the cervical segment (17), retinal slip (18, 19), or as an anticipatory mechanism driven by the efference copy (9, 13). We demonstrated that the latency and amplitude of 1st corrective saccade are closely correlated with the VOR gain during passive HITs. Thus, the extent of vestibular impairment and vestibular compensation may be crucial for the generation of corrective saccades during active HITs. It accords well with the finding that they closely correlate with dynamic visual performance in chronic vestibulopathies (8, 9). Indeed, these covert saccades during active head movements help stabilize gaze and aid functional recovery in patients with deficient vestibular function (17).

### Factors associated with the generation of corrective saccades during active HITs

Notably, no correlation was found between the latency and amplitude of 1st corrective saccade and the peak head acceleration during active HITs. Previous studies have examined only those with high-acceleration head rotations that closely approximated the template of head acceleration during passive HITs (4, 8, 9). By achieving these high-acceleration rotations, the inhibitory contribution from the contralateral side is silenced (20, 21). Our study had relatively lenient inclusion criteria compared to prior studies regarding head acceleration, because rapid head rotation was challenging for most patients during the acute stages of a vertigo spell. In addition, high head acceleration is difficult to generate voluntarily in elderly patients with musculoskeletal comorbidities. Nevertheless, corrective saccades consistently occurred even in patients with low head acceleration (< 1,500°/s^2^) in our study. Our findings suggest that active HITs can be employed in patients who are not able to elaborate ideal rapid head rotation voluntarily.

### Clinical implication of active HITs

Active HITs may be utilized for the virtual assessment of the HITs. Since the introduction of the ‘HINTS’ protocol (22), HITs have been widely used in acute vestibular syndrome (AVS). Nevertheless, HITs are heavily reliant on the expertise of the examiner, and it necessitates at least 100 tests per examiner for accurate data acquisition (23). Delivery of fast, unpredictable head impulses is crucial, and care must be taken to avoid artifacts (3, 24), which can hinder the popularization of HITs among primary physicians. Indeed, this challenge can hinder the device-based physiological triage of AVS in the emergency setting (25). This can also be a major hurdle to the broader application of “virtual HINTS” as an extension of the virtual neurologic examination for acute dizziness via a telemedicine platform. Spontaneous or gaze-evoked nystagmus can be captured at a high enough video frame rate of more than 60 fps (26, 27). Virtual alternate cover test can detect skew deviation by monocular occlusion of each eye with the patient’s palm for 2–3 s at a time. Meanwhile, application of active HIT and utilizing it as a telemedicine platform can have difficulty in two ways. For the patient, it is important to obtain high-speed head impulses, as vestibular hypofunction can be masked at low head velocity (13). A brief, instructive demonstration by the examiner could help ensure an ideal example with adequate size and speed of the head impulses (28). Additionally, our findings suggest that acute peripheral vestibular imbalance can be detected even in patients with relatively low head acceleration (< 1,500°/s^2^). For the examiner, the utility of active HITs mostly relies on catching the early, covert saccades during active rotation, which are difficult to discern by an untrained, naked eye on bedside examination. These early corrective saccades mostly arise during head movements and are mixed up with the VOR response, inflating the VOR gain. In this regard, a mobile-based quantitative measure of active HITs requires autonomic saccadic detection, which warrants a future study. The application of active-HITs and further virtual HINTS can greatly aid AVS triage in the near future. Implementation of a video application can facilitate both synchronous (online with patient and doctor interacting simultaneously) and asynchronous (offline evaluation of patients’ videos by doctors retrospectively) encounters (29). Remote evaluation can be conducted either via a separate facility or using patients’ smartphones or tablets via a mobile application (30). Our findings provide a preliminary clue that active HIT can also be efficiently implemented in virtual HINTS.

### Caveats and limitations of our study and suggestions for future research

Our study has several limitations. First, because of its preliminary nature, the sample size was small, which may lead to overfitting in estimating the diagnostic yield of active HITs. Relatively small and late corrective saccades can also be observed in healthy participants. Additionally, the eligibility criteria are rather lenient, with symptom onset within 7 days, outside the window to apply HINTS. Age also did not perfectly match that of the healthy participants. Indeed, VOR gain is mostly preserved or marginally decreased, yet corrective saccades tend to appear more frequently with aging (31, 32). Although internal validation replicates the robustness of active HIT parameters, our result remains vulnerable to overfitting. The exceptionally high diagnostic performance observed, with AUC values reaching 0.971, should be interpreted with caution. Despite the use of internal 5-fold cross-validation, these results remain susceptible to overfitting due to the relatively small sample size. Consequently, the specific cut-off values identified (latency ≤ 242 ms and amplitude ≥ 8.15°) require external validation in larger, independent cohorts to confirm their clinical robustness. Second, the lack of counterbalancing in the test sequence (where passive HITs always preceded active HITs) represents a significant limitation. This design was selected to help participants use as a reference when conducting self-generated HITs. Patients in real-world settings may have difficulty performing self-generated HITs. This issue may be particularly relevant when active HIT is implemented in a telehealth setting. As stated, an instructive demonstration or video demonstration may help mitigate this, but the diagnostic yield of active HIT without direct examiner demonstration may be lower than that observed in the present study. Indeed, this fixed order may have introduced a prediction bias or learning effect, potentially shortening the saccadic latencies observed during the subsequent active trials. Future research should utilize a randomized order to isolate the pure active response from the effects of prior stimulus exposure. Third, we did not analyze active HITs in the vertical canals due to artifacts and failure to maintain stimulation angle during HITs, which would limit the generalizability of our findings to HCs only. Given that abnormal results can be anticipated in the vertical canals in peripheral (33, 34), as well as in central vestibulopathy (35–37), refined data acquisition may also provide a diagnostic clue for patients with AVS. Fourth, analysis of each individual impulse was required to detect the covert/overt saccades in this study. Therefore, a processing algorithm is necessary to automatically filter, desaccade, and calculate the VOR gain for generalization. Our findings suggest that corrective saccades in healthy participants exhibit small amplitude and prolonged latency. Thus, automated detection of short-latency, large-amplitude saccades should be considered to detect vestibular imbalance during active HITs.

Despite these limitations, our study shows that detection of short-latency (≤ 242 ms) and large (≥ 8.15°) corrective saccades may provide a clue for detecting AUVP/VN during active HITs. Our findings may help the use of active HIT in patients presenting with acute spontaneous vertigo/dizziness, which warrants validation in future studies.

## Data Availability

The raw data supporting the conclusions of this article will be made available by the authors, without undue reservation.
