# Supplementary material for: Active head-impulse tests may help detect acute unilateral vestibulopathy: a preliminary study
Source: Front Neurol. 2026 Aug 19;17:1870852. doi: 10.3389/fneur.2026.1870852 (PMC13533766; doi:10.3389/fneur.2026.1870852)
Supplement: Supplementary file 1 [file Table_1.docx]

**Supplementary Table 1.** Internal validation of the discriminatory performance of active and passive HIT parameters for AUVP/VN

| **Parameters** | **AUC (95% CI)** | **5-fold cross-validated AUC (95% CI)** | **Cross-validated Sensitivity (95% CI, %)** | **Cross-validated Specificity (95% CI, %)** |
| --- | --- | --- | --- | --- |
| **1^st^ saccadic latency (active), ms** | 0.907 (0.811–0.979) | 0.889 (0.786–0.972) | 83.3 (64.1–93.3) | 77.3 (56.6–89.9) |
| **1^st^ saccadic amplitude (active),** ° | 0.971 (0.926 – 0.997) | 0.965 (0.917–0.995) | 87.5 (69.0–95.7) | 83.3 (66.4–92.7) |
| **1^st^ saccadic latency (passive), ms** | 0.949 (0.88 – 0.997) | 0.942 (0.854–0.997) | 79.2 (59.5–90.8) | 92.9 (77.4–98.0) |
| **1^st^ saccadic amplitude (passive),** ° | 0.989 (0.963 – 1.000) | 0.983 (0.946–1.000) | 87.5 (69.0–95.7) | 93.3 (78.7–98.2) |
| **VOR gain (passive)** | 0.897 (0.774 – 0.993) | 0.896 (0.762–0.996) | 79.2 (59.5–90.8) | 96.7 (83.3–99.4) |

* Cross-validated sensitivity and specificity were calculated from pooled out-of-fold classifications, and their 95% confidence intervals were estimated using the Wilson score method.
